# Barriers and facilitators of effective self-management in asthma: systematic review and thematic synthesis of patient and healthcare professional views

**DOI:** 10.1038/s41533-017-0056-4

**Published:** 2017-10-09

**Authors:** Clare Miles, Emily Arden-Close, Mike Thomas, Anne Bruton, Lucy Yardley, Matthew Hankins, Sarah E. Kirby

**Affiliations:** 1 Academic Unit of Psychology, University of Southampton, Highfield, Southampton, SO17 1BJ UK; 2 Department of Psychology, Bournemouth University, Poole, UK; 3 Primary Care and Population Sciences, University of Southampton, Southampton, UK; 4 NIHR Southampton Respiratory Biomedical Research Unit, University of Southampton, Southampton, UK; 5 Faculty of Health Sciences, University of Southampton, Southampton, UK; 6 Patient-Centred Sciences, Mapi Group, London, UK

## Abstract

Self-management is an established, effective approach to controlling asthma, recommended in guidelines. However, promotion, uptake and use among patients and health-care professionals remain low. Many barriers and facilitators to effective self-management have been reported, and views and beliefs of patients and health care professionals have been explored in qualitative studies. We conducted a systematic review and thematic synthesis of qualitative research into self-management in patients, carers and health care professionals regarding self-management of asthma, to identify perceived barriers and facilitators associated with reduced effectiveness of asthma self-management interventions. Electronic databases and guidelines were searched systematically for qualitative literature that explored factors relevant to facilitators and barriers to uptake, adherence, or outcomes of self-management in patients with asthma. Thematic synthesis of the 56 included studies identified 11 themes: (1) partnership between patient and health care professional; (2) issues around medication; (3) education about asthma and its management; (4) health beliefs; (5) self-management interventions; (6) co-morbidities (7) mood disorders and anxiety; (8) social support; (9) non-pharmacological methods; (10) access to healthcare; (11) professional factors. From this, perceived barriers and facilitators were identified at the level of individuals with asthma (and carers), and health-care professionals. Future work addressing the concerns and beliefs of adults, adolescents and children (and carers) with asthma, effective communication and partnership, tailored support and education (including for ethnic minorities and at risk groups), and telehealthcare may improve how self-management is recommended by professionals and used by patients. Ultimately, this may achieve better outcomes for people with asthma.

## Introduction

Self-management is an established, effective and guideline-recommended approach to controlling asthma.^1^ It has been defined by the US Institute of Medicine as “the tasks that individuals must undertake to live with one or more chronic conditions. These tasks include having the confidence to deal with medical management, role management and emotional management of their conditions”.^2^ With regard to asthma control, this encompasses adherence to treatment. Adherence enables individuals to self-manage their condition and is essential to the success of self-management interventions.^3^ Effective self-management has resulted in improved quality of life and reduced healthcare utilisation, days absent from work or school, and nocturnal asthma.^4^ However, despite effective medication being available, asthma is poorly controlled in over 50% of cases,^5,6^ and the promotion, uptake and use of self-management among people with asthma, carers of children with asthma, and healthcare professionals remain low.^7–9^ To maximise the benefits of self-management, barriers and facilitators to effective self-management (which may be encountered by the individual with asthma (or carer), the healthcare professional, or at the organisational level)^10–12^ need to be identified. Further, more effective treatment and management strategies are needed. Identification of the needs, beliefs, and behaviours of these individuals and organisational features^12^ can indicate where improvements should be focused to help groups of people least likely to benefit from existing self-management interventions, and potentially inform the design and implementation of future interventions.^10^

Quantitative reviews in this area have focused on identifying and comparing combinations of effective features of self-management interventions, and comparing methods of delivering and implementing these interventions.^4,8,12–18^ However, they contribute less to our understanding of the barriers and facilitators to self-management.^5,15,16^ Qualitative research, however, can provide in-depth information about behaviours, beliefs, emotions and relationships that may influence uptake of and adherence to self-management. Two qualitative reviews have synthesised the literature in this area.^19,20^ highlighted factors that can contribute to low acceptance of or adherence to self-management programmes for asthma, at the patient and programme level. They also identified the need for healthcare professionals to incorporate patient input in the development of treatment plans; to agree upon treatment goals; and to acknowledge patient knowledge of asthma based on personal experience. However, they were both relatively narrow in scope; the first review focused on adherence to medication, based on patient viewpoints only, and the second review focused specifically on barriers to action plan use. Thus, no qualitative review to date has encompassed the full range of barriers and facilitators to asthma self-management. The aim of our systematic review is to identify individual patient, professional and organisational barriers and facilitators to asthma self-management, by examining qualitative evidence from the perspectives of patients, carers and healthcare professionals.

## Results

### Study characteristics

**Fig. 1:**
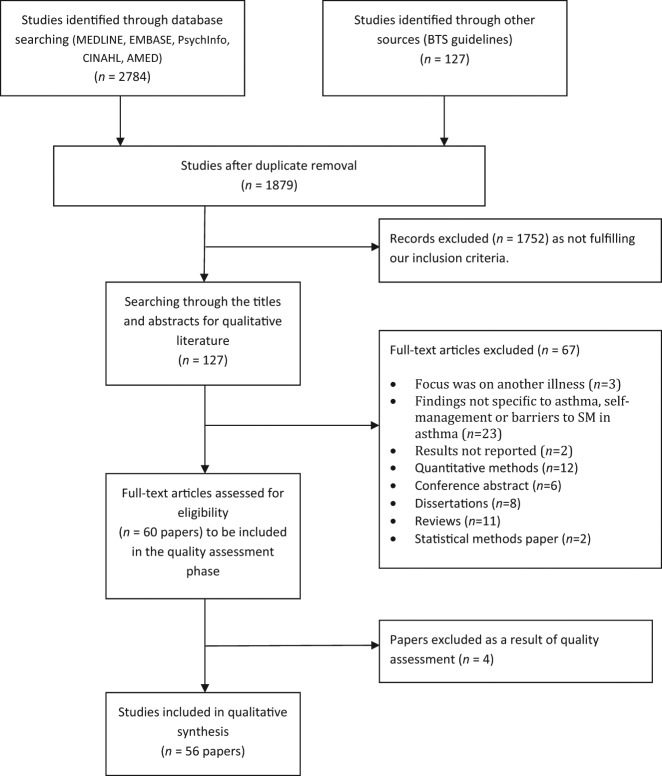
Flowchart of search process

The search results (Fig. 1) identified 2784 papers, of which 127 were deemed potentially relevant. Following application of exclusion criteria and quality assessment, 56 papers were eligible for the review. The 56 papers included in the review were published between 1997 and 2017, although two thirds of this research was published within the latter 10 years, reflecting a growing interest in this area, particularly within the USA. The majority of the research was conducted in the USA (*n* = 23)^21–43^ or the UK (*n* = 12),^11,44–54^ with fewer studies being conducted across the rest of the world: Australia (*n* = 6),^55–60^ Canada (*n* = 3),^61–63^ Taiwan (*n* = 3),^64–66^ Denmark (*n* = 2),^67,68^ Singapore (*n* = 2),^69,70^ Netherlands (*n* = 2),^71,72^ Germany (*n* = 1),^73^ New Zealand (*n* = 1),^74^ and Thailand (*n* = 1).^75^ Data collection methods primarily comprised interviews (*n* = 35)^23–26,29,30,35–38,41,44–53,55–58,60–62,64–66,68,72,74,75^ and focus groups (*n* = 21).^11,22,28,31,33,34,39,40,42,43,45–47,58,59,63,67,69–71,73^ A few alternative methods were also used: diary or journal data (*n* = 2),^21,27^ online free text responses (*n* = 1),^54^ and the recording of clinical consultations (*n* = 1).^32^ The following groups of participants were studied: adults with asthma (*n* = 25),^21–25,32,35,38–41,43,44,49,50,54–57,59–63,73^ children, adolescents and/or carers (*n* = 29),^11,26–31,33,34,36,37,39,42,47,48,51–53,62,64–68,70–72,74,75^ healthcare professionals (*n* = 9),^11,31,32,39,46,52,63,69,74^ and one study included school staff.^31^ There has also been a move over time to explore in more detail the views of minority ethnic and other at risk groups. These included African Americans (*n* = 6),^33–35,37,38,43^ South Asians (*n* = 2),^44,45^ Puerto Ricans (*n* = 1),^28^ Mexicans (*n* = 1),^30^ Latinos (*n* = 1),^41^ older adults (aged 50 and above; *n* = 1),^57^ those on a low income (*n* = 5),^21,23,25,51,65^ those from urban areas (*n* = 5),^21,23,33,34,51^ and those from rural areas (*n* = 2).^25,42^ Two studies focused on those with intellectual disabilities (*n* = 1),^60^ and low health literacy (*n* = 1).^38^ A subsection (*n* = 8) examined perspectives on use of various ways to deliver self-management interventions, such as within schools, or using mobile phones, patient advocates, pharmacist-delivered interventions, internet-delivered interventions, and by enhancing information given to HCPs before clinical conversations.^21,25,31,32,47,58,67,68^

### Thematic synthesis

**Fig. 2:**
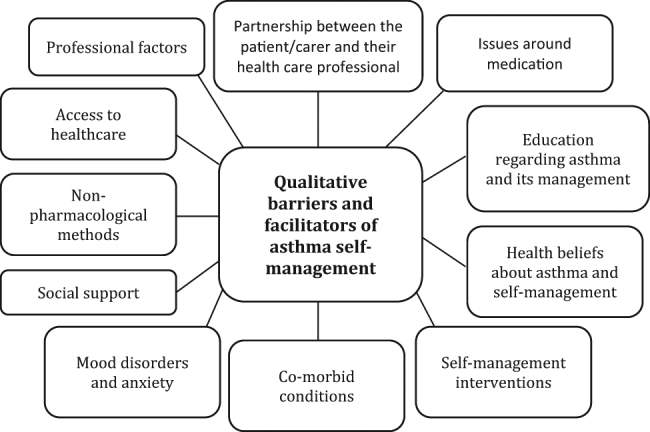
Themes identified during thematic synthesis

Thematic synthesis identified 11 main themes, within which analytic themes were identified that encompass the barriers and facilitators to asthma self-management found in this review. A diagram of the themes is presented in Fig. 2, and they are detailed in Tables 1–6. Barriers and facilitators to asthma self-management in relation to the themes are summarised below, and presented in Table 7.

**Table 1:** Partnership between patient and/or carer and HCP

Sub-theme	Summary of findings^a,b^
Communication between HCP and patient	• There is a perceived need for better communication between patient and HCP; for the patient to be included/acknowledged in the consultation; and for the patient to have a respectful relationship with their GP^22,24,29,38,44,54,61–63,73,74^ (P/Ca/HCP).
	• Patients who have a mutually trusting relationship with their HCP have confidence in their own understanding of asthma, avoid ED re-attendance^44^, and are more likely to adhere to SM advice^61^ (P).
	• Patients want GPs to take an interest in them and try to understand their experience^38,54,59^ (P). Some GPs need to learn about patients’ personal circumstances by listening to the patient, in order to develop a relationship and improve medication taking behaviour^21,62^ (P/ R).
	• GPs value and seek acknowledgement for the skills they use in persuading patients to take medications properly^46^ (HCP).
	• Some people with intellectual disabilities are frustrated when HCPs talk to their carers rather than directly to them^60^ (P)
	• Some carers value nurse communication with their children and intervene in consultations between their child and HCP to clarify information provided by the child (Ca). Nurses want to hear from the child, not just the carer during consultation^52^ (HCP).
	• Non-focused questions give more opportunity for children to influence the agenda of consultations but children find it easier to respond to focused questions^52^ (R).
	• Patient advocates can sometimes facilitate communication through interactions with patients^21^ (P).
	• Some HCPs aim to be empathic, non-judgmental and open; putting themselves in the patient’s shoes^63^ (HCP)
Continuity and consistency of care	• There is a perceived need for continuity of care to avoid patient and carer frustration and assessment difficulties for clinicians and to develop an effective patient-GP partnership^22,24,29,34,59^ (P/Ca).
	• HCPs do not always give consistent advice regarding treatment decisions, and are not always consistent in tailoring medication to the patients’ or carer’s routines^24,74^ (P/Ca).
	• If the patient/carer perceives inconsistent advice is given by different GPs or by the same GP on different occasions, they can become confused. This might lead to patients being unlikely to go to the GP for future help^74^ (P/Ca).
	• Some HCPs use strategies to prevent patients re-attending the ED, such as: targeting high risk patients; offering ‘privileged access’; and providing out of hours continuity of care^44,63^ (HCP).
Patient/carer perception of GP care as ineffective or inadequate	• Some patients, particularly South Asian, African American, and low health literate patients and carers, feel their GP or other primary care providers do not effectively treat them^38,43,74^ and view care as substandard. Some perceive that treatment is quicker and more efficient through the ED, rather than through their GP and report being more satisfied with advice given by nurses, A&E doctors, and pharmacists^37,45,74^ (P/Ca).
	• When treatment is not perceived as effective and subsequent changes in treatment are also not deemed effective, patients sometimes become non-compliant with their treatment and avoid asthma management advice^50^ (R).
	• HCPs are reported as being good at dealing with symptoms but some patients expect them also to locate the cause of asthma and would prefer longer consultation times^43,48,73^ (P).
	• Some carers have a lack of confidence in the GP’s advice and search among different doctors to find an effective way to manage their asthma^65^ (Ca), particularly as some GPs are seen as unhelpful, and not providing enough information to patients^45^ (P).
	• Children are concerned that medications do not work.^26^ Young people and adolescents, particularly African American adolescents, do not perceive the need for regular, routine health care visits^33,73^ (Ch/P).
	• Some carers perceive there is reluctance for GPs to prescribe steroids when it is necessary^74^ (Ca) and that diagnosis is not accurate, diagnosing a less severe condition, e.g., coughing being attributed to an ear infection rather than asthma^29^ (Ca).
	• Carers who stop their children taking medications due to perceived ineffectiveness still visit the hospital for asthma attacks^75^ (Ca/R).

^a^ *SM* self-management, *GP* general practitioner, *ED* emergency department, *CAM* complementary and alternative medicine, *HCP* health care professional, *WAAP* written asthma action plan, *PEF* peak expiratory flow

^b^ Type of person who expressed their viewpoint (*P* patient viewpoint, *HCP* health care professional viewpoint, *Ca* carer viewpoint, *Ch* child/adolescent’s viewpoint, *R* researcher’s viewpoint)

**Table 2:** Issues around medications

	Summary findings^a^
Concerns over safety and side-effects of asthma medicines	• Some patients and carers believe asthma medications are unsafe, have side effects, decrease in effectiveness over time, and lead to long-term dependence^23,28–30,32,36,40,48,62,64,66,72–74^ (P/Ca/Ch). Despite this, patients and carers believe asthma medicines are necessary^62,68^ (Ca).
	• Patients have most concerns about steroids, due to side effects such as weight gain^24,34,54^ (P). HCPs believe short-acting beta-2 agonists (SABAs) are overused, but some patients and carers, including adolescents and those from ethnic minorities and with intellectual disabilities, are reluctant to use ‘too much’ medication, viewing them as “toxic”, and fearing tolerance and addiction might develop^23,30,32,33,36,60,73^ (P/Ca/Ch).
	• Some older patients with a longstanding diagnosis of asthma were reluctant to use their reliever medication due to side effects (tremor, palpitations) or believing their symptoms were not bad enough to warrant use^57^ (P).
	• Some patients are reluctant to use medication on a regular basis, so use preventative medicines inconsistently^24,68^ (P).
	• CAM medications are viewed as safe whereas conventional medicines are viewed as unsafe. CAM should be used alongside rather than instead of conventional medication^23^ (P).
Overprovision of asthma medications from the HCP	• Some patients and carers are reluctant to follow GP treatment plans that include high dosages of preventer medications^21,28,48,52,74^ (P/Ca). They believe high dosages have limited benefits and are not useful^21,48^ (P/Ca).
	• Some carers do not like GPs experimenting with dosages that involve increases^75^ (Ca).
	• If patients perceive their doctor’s plan to be “too drastic” (i.e., if they view a prescribed dose of prevent medication as too high, or advice from the HCP to seek emergency care as unnecessary), they adapt their own plan, based on their own experience of dealing with symptoms^55,72,73^ (P).
Practical barriers to medication adherence	• Patients perceive inhalers as time-consuming to use^22^ and sometimes forget to take medications away on holiday. As a result asthma symptoms worsen^24^ (P).
	• Adolescents are reluctant to use inhalers if they have to leave class, take the inhaler without sufficient privacy or in front of strangers^36^ (Ch).
	• Some patients report problems accessing medications, due to costs of medications, insurance coverage, problems obtaining refills at the pharmacy, and having to travel long distances for medications^36,39,41,43,73^. Some patients believe medicines are dispensed with incorrect instructions or medications by the pharmacist^22,24^ (P).
	• Costs of medication, not taking medication as prescribed, and not attending a medication review are potentially preventable factors that lead to ED re-attendance^56^ (R).
	• Some carers/ patients were too busy to remember to use PEF^65^ or medications^24,29,72^, and some parents skipped doses to avoid upsetting their child^72^ (Ca/P).
	• Although health care providers feel they are clear about the differences between controller and rescue inhalers, many patients do not know the difference. Some older adults and adolescents misunderstand how often to use controller medication^39,40,42^ (HCP/P).
	• Some adolescents, and carers including school staff report difficulties in administering medications during school hours^28,31,34,42^ (Ca).
	• Patients, including those with intellectual difficulties, reported physical difficulties using some devices and inhalers^39,60,73^ (P).
Trial and error approach to medication management	• Some patients and carers use a trial and error approach to taking their medications (by stopping or reducing medications). They test whether they still need medications and adjust medications according to symptoms^11,22,29,51,75^ (P/Ca).
	• Some patients see this experimentation as ‘not bothering’ the doctor^11^ (P).
	• Patients, including african american young adults and carers who perceive themselves/their child as not having asthma typically use this approach, reducing their medication when symptoms improve^30,43,50^ (P/Ca).
	• Trial and error can lead to worsening of symptoms, causing patients to perceive that preventative medicines are not necessary^11,22^, or effective^54^ (P).
	• Trial and error approaches are based on health beliefs and past experience, and can occur in collaboration with the GP, increasing patients’ confidence in self-managing their asthma^40,42,57^ (P/Ca). Professional advice is tested and adapted to fit in with patients’ and carers’ understanding and beliefs of whether symptoms are ‘tolerable’ ^51,52^ (P/Ca).
	• Some carers use trial and error approaches to decide whether their children need to continue taking inhaled corticosteroids^51^ (Ca).
Reasons for/against CAM use	FOR:
	• Patients who were more interested in and positive about CAM tended to be female^73^ (P).
	• Many patients and carers use CAM to be in control of medication rather than feeling dependent on it^49,56,75^ (P/Ca).
	• Those who use CAM believe it gives them a more tailored treatment approach that is: 1) effective, natural and non-invasive, 2) good for mild symptom control, and 3) safer than conventional medicines^23,48,66^ (P).
	• Many CAM users believe the combination of CAM and Western medicine is superior to either approach used alone^73^, as it results in improved effectiveness of rescue bronchodilators^22^ (P).
	• Some carers believe CAM improves immunity^64,65^ (Ca).
	AGAINST:
	• Some patients think CAM medicines are ineffective in managing asthma, (i.e., ‘severe’ symptom control) and there is a lack of scientific evidence recommending many of them^45,48^ (P).
	• Some CAM approaches were strongly advocated, but were also labelled as too time consuming^73^ (P).
Preference for medications by patients	• Some carers prefer their child to take a particular type of medicine (e.g., nebuliser or the metered dose inhaler)^29^ (Ca).Carer and patient preferences were often based on habit, method of administration they felt most effective, confusion surrounding particular medications, side effects perceived and fear of taking too many medications^26,30,74^ (Ca/P).
	• Carer and patient preferences were often based on habit, method of administration they felt most effective, confusion surrounding particular medications, side effects perceived and fear of taking too many medications^26,30,74^ (Ca/P).
Facilitators of medication use	• A number of strategies were used to remember when to take medications, including visual cues, reminders, setting phone alarms, and matching to routine daily events^40,62,72^ (P/Ca). This was particularly the case for those with intellectual disabilities and caregivers of African urban teens ^34,60^ (P/Ca). A proactive attitude was also a facilitator^62^ (P).
	• Adolescents are more likely to use inhalers if it is nearby, and they are able to use it without disrupting activities, with sufficient privacy, and with support from friends, teachers and coaches^36^ (Ch).

^a^ Type of person who expressed their viewpoint (*P* patient viewpoint, *HCP* health care professional viewpoint, *Ca* carer viewpoint, *Ch* child/adolescent’s viewpoint, *R* researcher’s viewpoint)

**Table 3:** Education regarding asthma and its management

Sub-theme	Summary findings^a^
Adult patients and HCPs indicate or express a need for education in asthma SM	• There is a perceived need for patient education in asthma SM. Many patients lack understanding of their medications, have low asthma knowledge scores, and do not know why they should monitor PEF. Many want more information about asthma and would be willing to participate in research to learn more about their asthma^21,22,24,44,45,55,56,62,68,73^ (P). Patients would also like to be shown how to use their inhalers effectively^39,62,73^ (P).
	• Low patient asthma knowledge score is a potentially preventable factor leading to ED re-attendance^56^ (R) and education is needed around the importance of using controller medication^39^ (R) and understanding of asthma control^40^ (R). Explanations and information needed to be adapted to the patient’s needs^43,62^ (P).
	• Some HCPs (nurses and GPs) report feeling they are not given sufficient training in written action plan use^69^ (HCP).
	• Asthma related education is one possible approach to dealing with patient non-adherence with treatment plans^46^ (R).
	• Some with severe asthma view the use of information booklets, solely to convey health information, as inadequate, with the information being directed towards a “moderate” type asthmatic^61^ (P).
	• Brief printed information and oral information from patients’ doctors was the preferred method of education. In-patient rehabilitation programmes were criticised by patients as lacking peer group support, and only being suitable for children and older adults^73^ (P).
	• Patient advocates^28^, pharmacist educators^45,58^ and nurses^52^ can help reinforce SM education^21^ (R).
Carers, their children and schools need education in asthma and SM	• Some carers and their children lack understanding of asthma and asthma control and are confused about asthma medications^26,29,42,47,65^ (Ch/Ca). Occasionally carers do not feel competent or confident in administering medications to their children^75^ (Ca).
	• Some carers feel they are given insufficient information to manage the asthma^75^ and believe that after education they could further benefit from review and reinforcement^29^ (Ca).
	• Some children understood the importance of medications, although they were not always able to distinguish between different types of medications. Younger children described medications in terms of side effects or taste^26,28^ (Ch/R).
	• In some young people with severe asthma, asthma camps and the use of multiple educators were effective in learning about asthma management^27^ (R, Ch).
	• Education prevents unnecessary ED re-admittance^65^ (R/ Ca).
	• Some carers provide instructions for teachers to manage their child’s asthma during school hours^64^ (Ca) and carers and adolescents were concerned that staff have a lack of knowledge about triggers and the seriousness of symptoms^34,39,42^ (Ca). At present the responsibility for educating teachers in asthma SM falls on the carer^64^ (R).
	• School staff report that parents/carers do not provide the school with the child’s asthma action plan^31^ (S), and it was unclear as to what school staff, school nurses, physicians, parents and children were allowed to do and when^31,39^ (Ca / HCP / S). Urban African American adolescents report that teachers do not believe them when they report having asthma symptoms^33,34,37^ (Ch/Ca).
Patients with low health literacy, intellectual disabilities, ethnic minority patients & their carers indicate or express a need for education in asthma SM	• Reports of poor understanding of asthma medications may be more widespread in South Asian patients than white patients^44,45^ (R).
	• South Asian patients use less corticosteroid during an attack and fewer preventative medicines (possibly due to a lack of understanding regarding preventative medicine), compared to white patients^44^ (R). They express a need for written information^45^ (P).
	• Some HCPs felt language barriers prevented them from educating ethnic minority patients, or patients not fluent in their language^62,69^ (HCP).
	• Patients with low health literacy, African American young people, adolescents and their carers reported the need and desire for accurate knowledge relating to asthma^37,38,43^ (Ca/Ch/P). Inaccurate medical knowledge leads to insufficient care^37^ (R). Carers of urban African American adolescents were concerned that the adolescents needed to know how to respond to an ‘asthma crisis’ independently^34^ (Ca).
	• Mexican and Taiwanese mothers, and patients with low health literacy reported little knowledge of asthma^30,38,66^, obtained mainly from lay sources^30,38^ (Ca). Some patients with intellectual disabilities reported not being shown how to use their inhaler devices correctly^60^ (P).
	• South Asians who were educated in using action plans became confident in and receptive to using them to manage their asthma^45^ (P).
Recognising symptoms and awareness of triggers	• Some patients do not use their action plans if they are not confident about recognising their symptoms^55,56^ (P).
	• Some patients and carers report difficulty in distinguishing asthma symptoms from a common cold^70^ (Ca).
	• Most patients actively avoid their triggers, and acknowledge the importance of this in managing their asthma^11,22,47,75^ although some cannot identify their triggers^22,26,45^ (P).
	• Some adults with asthma viewed the awareness of triggers as a highly personalised responsibility and difficult to generalise, believing it was therefore not essential to work in partnership with their HCP ^59^ (P/R). This view was shared by urban African American adolescents^33^ (Ch).
	• Not being able to recognise asthma symptoms is a potentially preventable factor that leads to ED re-attendance^56^ (R).
	• Good recognition of symptoms was associated with education regarding asthma. e.g., children learning about asthma symptoms from a school project and frequent education from their HCP^27^ (Ch, R).
	• Some adolescent, Latino, and older adult patients recognise limitations in their ability to control all environmental triggers, e.g., weather changes^27,40,41^ (Ch).

^a^ Type of person who expressed their viewpoint (*P* patient viewpoint, *HCP* health care professional viewpoint, *Ca* carer viewpoint, *Ch* child/adolescent’s viewpoint, *R* researcher’s viewpoint, *S* school personnel’s viewpoint)

**Table 4:** Health beliefs about asthma and its management

Sub-theme	Summary findings^a^
Health beliefs of patient or carer, as a barrier to SM.	• Patients’ health beliefs and illness representations^30^ can be a barrier to care and SM, directly influencing how they manage asthma^24,28,32^ (R).
	• Patients often omit health beliefs that are not consistent with western medicine from discussion with their GP^24^ (P).
	• Patients who have confidence in taking medicines also avoid ED re-attendance^44^ (R).
	• Some ethnic minority patients and carers (Puerto-Rican patients, and Mexican mothers) treat asthma based on beliefs that they need to address imbalances between hot and cold^28,30^ (Ch/Ca). They also focus on self- management techniques that alter the environment or emotions^28^ (Ch/Ca).
	• Some children and carers attribute their asthma to having too much exercise and can list some environmental triggers^26,66^ (Ch/Ca). Others (including African American women, children and carers) reported physical activity to be beneficial^33–35^ (P/Ca/Ch)
	• Many young people assess asthma in terms of how ‘normal’ they appear in front of their peers^36,51^ (Ch).
	• Many carers (including Taiwanese mothers) use the occurrence of asthma attacks, symptoms and behavioural change to assess the asthma^51,66^ (Ca).
	• The extent to which asthma symptoms impact on the family is used to assess severity of asthma^28^ (Ca).
Validity of the diagnosis and acceptance	• Some patients do not accept their diagnosis and consequently have poor self-management. They may deny their asthma or minimise its severity^23,53,62^, forget medications^29^ and not follow action plans^55^ (P).
	• Some carers from ethnic minorities believe the disease is only present when their child is symptomatic^30^ (Ca).
	• Text message mobile technology might help some patients accept and come to term with their diagnosis^47^ (P, HCP).
	• Some carers find it difficult to accept the diagnosis, due to negative stigma^74^ and no clear diagnostic test^52,75^ (Ca).
	• Some carers avoid admitting to their child’s diagnosis and describe the difficulties in diagnosing asthma^30,65^ (Ca).
Views of asthma with regards to self-management (positive and negative aspects)	POSITIVE:
	• Most patients are aware of the episodic nature of asthma^30,45,67,68^ and the main aims and components of self-management^59^ (P).
	• Due to the potential negative impact on self-image, some patients are motivated to fight back and control their asthma^49^ (P). Disliking feeling out of control with symptoms is a motivator for gaining and maintaining control^22,67^ (P).
	• Most carers want their children to be treated normally and not let the asthma limit their children’s lives. Carers do not want asthma to be used as an excuse to not do particular things, i.e., chores^64^ (Ca).
	• Some older patients (>50 yrs), with a recent diagnosis seek to understand the cause of their asthma and access information to self-manage it^57^ (P).
	NEGATIVE:
	• Some patients view asthma as a burden^67^ (P).
	• A sense of despondency resulted from asthma attacks despite positive personal action^59^ (P).
	• Some patients, including those with intellectual disabilities are embarrassed to use inhalers in public due to concerns about what others will think^22,30,33,45,54^ (P/Ch).
	• Some patients tend not to disclose their asthma in public and prefer to describe their symptoms using terms such as ‘breathing difficulties’^45^ (P).
	• Some older patients (>50 years of age) with a long-term diagnosis, base their self-management strategies on past experience, e.g., concealing symptoms due to the negative stigma of asthma^57^ (P).
	• Some carers are concerned that asthma will affect learning and relationships^64^ (Ca).
Motivators of self-management	• Patients tend to be motivated to manage their asthma: i) when symptoms cause discomfort; ii) if they believe asthma may have serious consequences; and iii) when asthma affects a valued activity^30,49,63^ (P/HCP). Some were not motivated to act until it posed a life threatening state^59^ (P).
	• Some patients with severe asthma are motivated to manage their asthma by balancing good aspects of treatment (e.g., medicine helps them to engage in everyday activities), with bad aspects (e.g., side effects of medicines)^61^ (P).
	• Some teenagers, including Urban African Americans, do not see the importance of visiting the doctor for review,^11,71^ particularly when feeling well^33,71^ and can be unwilling to take medications^71^ (Ch).
	• Carers tend to focus on the most bothersome symptoms, so adapt their written action plan (Ca). Treating only symptoms that bother the carer, instead of self-adjustment of medication in line with action plans, goes against GP advice^70^ (R).
	• If asthma is normalised by the patient and its effects not noticed there is no motivation to self-manage^36,49^ (P/Ch).
Self-efficacy for self-management of asthma	• In adolescents poor asthma control is associated with limited perceived ability to control asthma^71^ (R).
	• High self-efficacy is associated with patient beliefs that exercise, trigger avoidance, using an inhaler, and taking preventer medication makes a difference^27^ (Ch/R), and that they have the skills knowledge and confidence to control things on a daily basis^59^ (P).
	• Low self-efficacy for self-management was influenced by factors out of the individual’s control (e.g., others’ smoking or the weather)^59^ (P).
Child takes responsibility for care in different ways to those expected by the parent	• Carers and children hold differing views of how to be responsible for managing their asthma^53^ (R).
	• Some children take responsibility for their asthma by making the effort to minimise the limitations of the illness and using non-medical interventions, such as sitting out an activity^42,53^ (R/Ch).
	• Some children report awareness of triggers and tell someone when they feel unwell^26^ (Ch).
Transfer of responsibility in managing asthma	• Transfer of responsibility from carer to child in managing asthma is gradual,^34,53^ and negotiated^29,34^ (Ca, R).
	• Text message interventions that help the patient monitor symptoms are useful to aid transition of responsibility^47^ (R).
	• Some carers secretly monitor children’s asthma symptoms and whether they are taking their medications^52,53^ (R, Ca).
	• Many Taiwanese carers are fearful when children start school, as they will be unable to manage their child’s asthma during school time.^64^ Others want the child to take responsibility for their self-care and medication,^64,66^ including when at school,^31^ and teach them to avoid asthma episodes in ways they perceive as effective, e.g., by dressing warmly and changing clothes when wet^64^ (Ca).
Who is responsible for managing asthma	• Many adults and carers believe that asthma self-management is their responsibility based on their own judgement and awareness of triggers, without an alliance with their GP^39,59,72^ (P/Ca).
	• Parents have concerns over balancing monitoring medication use and encouraging independence (feeling children should take responsibility in case they are not around during an attack)^28,52,53,66^ (Ca).
	• The primary carer usually takes responsibility for young children but parents expect older children to do so^29^ (Ca/R).
	• When children do not successfully manage their asthma, carers take this responsibility back^42,75^ (Ca/Ch).
	• Some nurses suggest involving children in consultations to show their carers they are becoming independent^52^ (HCP).
	• Children and Teenagers can have worse adherence and morbidity due to less parental supervision^28,72^ (Ca/R).
	• School staff are often unclear how to manage asthma, with some being over cautious (e.g., unnecessarily excluding African American teenagers from activities), or under cautious (e.g., not believing African American teenagers reporting symptoms)^33,34,37^ (Ch/Ca/S).
Goals of patient and treatment expectations	• Patients (including Urban African American adolescents and young adults) and carers main goals are to treat symptoms (rather than prevent symptoms or attacks)^33,43,62,68,72,74^ (P/Ca/Ch).
	• Some patients aim to live symptom-free,^67^ to be cured, or to have control over their asthma^49^ (P). Others aim to learn to live with asthma^30^ (Ca).
	• Few patients have a goal, have worked with the HCP to set a goal, or have planned ways to achieve a goal^22^ (P). Some HCPs suggest this is due to a lack of time necessary to carry out with patients^39^ (HCP).
	• Some patients expect treatment to improve their breathing and prevent further attacks^45^ (P).

^a^ Type of person who expressed their viewpoint (*P* patient viewpoint, *HCP* health care professional viewpoint, *Ca* carer viewpoint, *Ch* child/adolescent’s viewpoint, *R* researcher’s viewpoint, *S* school personnel’s viewpoint)

**Table 5:** Self-management interventions

Sub-theme	Summary findings^a,b^
Positive feelings over action plan or guideline use from patients and HCPs	• Some patients have positive attitudes to action plans^55,62^ and guidelines^46^ (P), and felt that regular reviews could facilitate their control and empower them to self-manage their condition^54^ (P). Some caregivers of urban African American teenagers instigated the development of an asthma action plan with their school nurse^34^ (Ca). Some adolescent patients also saw Internet action plans as useful^71^ (P/Ch).
	• Patients who do not have a plan report feeling it would be useful^45,55,62^ and some would like to try peak flow readings alongside their action plans^55^ (P).
	• Some GPs report that guidelines are useful for managing asthma, particularly for those with difficult/severe asthma and in deciding medication steps^46^ (HCP).
	• School nurses and staff wanted an action plan for all children with asthma, but did not receive one in most cases^31^ (S). Many HCPs plan ahead if they are expecting a child who will need school documentation including an asthma action plan^39^ (HCP).
Negative feelings over action plan or guideline use from patients and HCPs	• Some patients report not being given an action plan as a reason for not using one^55^ (P).
	• Some carers prefer their own judgement to using peak flow^51^ (Ca). Some adult patients reported peak flow monitoring to be ‘nonsense’ or ‘frightening’^73^ (P).
	• Adolescent patients do not use action plans if they believe their asthma does not need further management.^33,71^ Some are not willing to use electronic action plans that involve monitoring over long periods^71^ (Ch/ R).
	• African American women report that mood and memory problems can be a barrier to remembering to follow their asthma action plan^35^ (P).
	• Occasionally nurses believe action plans can be dangerous (for intelligent people) as patients may manage on their own for too long, not return for their review and so encounter severe difficulties^11^ (HCP)^c^.
	• Some GPs regard actions plans as irrelevant,^11^ impractical,^44^ and time-consuming.^69^ While they use action plans for other conditions, such as diabetes they do not use them for asthma^44^ (HCP).
	• Some GPs have reservations over using guidelines as they are not useful for medication adjustment and are concerned that they are based on out-of-date evidence^46^ (HCP).
	• Use of action plans by HCPs ranges from little use to most of the time^39,46^ (HCP/R), and some patients report having never seen or possessed a written asthma plan^39,73^ (P).
	• Despite attending regular asthma reviews, some adults with asthma were sceptical about the interest, knowledge, and understanding demonstrated by GPs^59^ (P).
	• Some patients and GPs have low self-efficacy for using action plans. This mainly applies to patients who have not accepted their diagnosis^55^ and GPs who infrequently prescribe action plans^69^ (P, HCP).
	• Some patients feel action plans need to be ‘modified’ in some way (unspecified), to meet the needs of those with severe asthma^61^ (P). Other patients feel that they can decide their own plan of action without consulting their GP for review until they decide they should^33,39,59,73^ (Ch/P), and perceive generic action plans as patronising or condescending^59^ (P).
	• Some HCPs from Singapore follow their own action plan, when they cannot find the standard action plan given to them in training^69^ (HCP).
Perceived relevance of action plans and guidelines for particular types of people	• Some patients think action plans are good for patients with asthma they perceive to be worse than their own, or for ‘others’, but not relevant to them personally^11,71^ (P).
	• Some GPs feel action plans are best for motivated^11,69^ or educated^69^ patients and not appropriate for newly-diagnosed patients, those already self-managing,^11^ those with poorly controlled asthma,^46^ non-compliant patients or patients that do not understand them^11,46^ (HCP).
	• Some HCPs feel that guidelines are only useful to newly qualified staff^46^ (HCP).
	• The amount of importance that carers place on consulting a GP depends on the carers' level of concern, and their confidence and experience in using a written action plan^70^ (Ca, R).
Positive views of interventions (Internet, text message, booklet/DVD and pharmacist telephone)	• Users reported being satisfied with an Internet intervention^68^ (P, HCP). Some patients saw it as useful for identifying triggers and monitoring symptoms^50^ (P). Some GPs believe it benefited their patients, in understanding asthma, reducing symptoms and improving compliance. They think it was also good for record keeping and performing calculations^68^ (HCP).
	• Some patients, including those with intellectual disabilities, liked the convenience of being monitored by a text message diary or mobile phone alarm, as it gives them a sense of control whilst being supported^34,60,67^ (P). Adults and Older Adults found it useful to create reminder systems and routines to facilitate medication use^40,62^ (P).
	• Some Urdu speaking patients view written information booklets on managing asthma and videos in Urdu as useful and helpful^45^ (P). Young African American patients felt that an online programme with email and text messages would be helpful and desirable^43^ (P).
	• Some patients reported that a pharmacist telephone intervention was helpful and positive in giving SM education, for asking questions, and for getting feedback^25^ (P).
	• Internet-based self-management interventions were viewed by patients as useful for identifying triggers and observing symptoms; instant feedback regarding lung function; and reacting to changes that occur in asthma status.^71^ Email communication and electronic consultations were viewed as useful^71^ (P/Ch).
	• Internet interventions were better accepted by those with poorly controlled asthma. This group was more willing to use an electronic action plan and was not concerned about the time taking to monitor symptoms^68,71^ (R).
Negative views of interventions (Internet, text message, booklet)	• Some patients react to alert messages to increase medications with disbelief and consequently do not adhere to taking increased dosages.^68^ They would like more information on side effects of medications^68^ (P).
	• Lack of confidence with computers can hinder use of Internet interventions by patients and GPs^68^ (P, HCP).
	• Patients gave feedback on what they did not like about a text message service (frequency and type of message), suggesting improvements to its design^67^ (P).
	• Although some patients with severe asthma value information gained from websites, lay sources, medical journals, and information booklets, some prefer information from a specialist, using down-to-earth language^30,60,61^ (P).

^a^ Interventions aside from taking medications and avoiding triggers (i.e., action plans, guidelines and research interventions)

^b^ Type of person who expressed their viewpoint (*P* patient viewpoint, *HCP* health care professional viewpoint, *Ca* carer viewpoint, *Ch* child/adolescent’s viewpoint, *R* researcher’s viewpoint, *S* school personnel’s viewpoint)

^c^ Note that this viewpoint may be out of date as the study was published in 2000^11^

**Table 6:** Remaining themes

Theme	Summary findings^a^
Co-morbidities	• Patients often report comorbidities, both related and not related to asthma, which they are managing alongside their asthma. Therefore, managing their Asthma may not be their top priority^21,24,35^ (P).
	• Asthma medications can be viewed as having an undesirable effect on other health conditions, and some patients will not adhere to asthma medication if they are on a number of medications for multiple conditions^35,40^ (P)
	• Patients were encouraged to engage in health lifestyles (e.g., weight loss) in order to benefit asthma as well as other conditions at the same time^32^ (HCP).
	• Comorbid conditions (e.g., mood/memory/pain) can constrain asthma management (e.g., by forgetting to take medications, or reducing physical activity). Asthma symptoms and management can also constrain the management of co-morbid conditions (e.g., corticosteroids slowing weight loss)^35^ (P).
Mood and anxiety	• Patients may have other stressors in their lives, such as employment, housing issues and difficulties with personal relationships that are barriers to effective SM^24^ (P).
	• Anxiety about asthma can reduce the emotional well-being of older adults with asthma^40^ (P).
	• Fear of deportation for patients from ethnic minorities can cause stress, which patients believe contributes to their asthma exacerbations^44^ (P).
	• Depression may lead to non-compliant behaviour. Asthma can lead to low self-worth and the patient feeling different to others, leading to withdrawal from society. They may neglect SM and their health deteriorates^50^ (R).
	• Many carers find it stressful managing their children’s asthma, particularly when the sole responsibility lies with the carer^30,64,75^ (Ca). Carers’ emotion influences the emotions of their children^75^ (R).
	• Transition of responsibility from carer to child can make the carer feel powerless in managing and controlling their child’s asthma^28^ and both carers and children fear severe and fatal asthma attacks^28^ (Ca/Ch).
	• All family members are stressed by asthma-related trips to A&E^65^ (Ca).
	• Patients are anxious about the possibility of having a severe asthma attack in public, with bystanders not knowing what to do^59^ (P).
Social support (positive influences)	• Those with asthma benefit from building a strong, emotional support network, so family and close friends may need to be included in educational interventions for asthma SM^22^ (R).
	• Family, friends and co-workers can help patients manage their asthma by reminding them to take medication, teaching them about asthma, keeping them calm during an attack, providing transport to appointments, not smoking inside the house, helping with domestic duties when symptoms are increasing and providing emotional support^22,28,30,33,34,42,44,45,48,60^ (P/Ch/Ca).
	• Patients and carers learn about asthma from other family members and social networks with the illness^27,37,41,48^ (P/Ca).
	• Patient advocates can provide social support to the patient^21^ (R).
	• The family adapts to accommodate asthma management^28,75^ (Ca).
	• Patients express a need for local support groups^59^ (P).
Social support (negative influences)	• Family members and significant others can upset those with asthma by their over- and under-reactions to the condition, and unhelpful behaviours (e.g., smoking indoors).^41,54^ They can nag about medication taking and disregard severe symptoms or be unwilling to talk about the illness after an attack.^22^ Family members can also warn patients of side effects of steroids, such as weight gain^45^ (P).
	• Some employers may decline to employ those with asthma if there is a history of absenteeism due to their asthma^45^ (P).
	• Some patients with asthma do not like to impinge on others lives^22^ (P).
	• Some South Asian patients may adopt a more passive approach to managing their asthma. Some white patients seem to take a more proactive approach to SM^44^ (R).
	• Some GPs face problems getting patients to comply with treatment when opinions from extended family and traditional healers conflict^74^ (HCP).
	• Some adolescents (including ethnic minorities) had experience of social rejection by teachers and peers who the adolescents feel disregard their asthma symptoms^31,33,34,37,71^ (Ch/Ca).
	• Some adolescents and young people can be reluctant to tell their friends about their asthma, preferring to use a need to know basis^33,43^ (Ch). Others were less/not concerned if their personal health was at risk^33,42^ (Ch).
Non-pharmacological methods	• Patients and carers use non-pharmacological methods to attempt to manage asthma symptoms before taking reliever medication. These methods include drinking water, tea or black coffee, lying down/resting, using relaxation and breathing exercises, taking a bath, inhaling steam or getting fresh air, fan use, topical chest ointments, and praying^30,32,33,35–37,39,41,54,73^ (P/Ca). Adolescents with uncontrolled asthma used more of these methods and delayed using mediation longer^36^ (Ch).
	• Preventative methods were used to avoid onset of asthma symptoms. These included complementary or alternative therapies (e.g., acupuncture), asking people to smoke elsewhere, opening windows, using a dehumidifier, and when cold using a scarf and prewarming the car^33,34,37,66,73^ (P/Ca).
	• Lifestyle changes were also used to attempt to improve asthma control. These methods include diet, weight loss, exercise, and smoking cessation^32,33,54,73^ (P).
Access to healthcare	• Some patients and carers (including latino and African American participants) report problems in accessing healthcare, including barriers such as costs of healthcare and insurance coverage, problems accessing medications (see Table 2), and time required for rehabilitation programmes or goal setting^22,24,39,41,43,54,73,75^ (P).
	• Some patients report difficulties getting appointments due to long waiting-times and unreturned phone messages^21,24,73^ (P). Patient advocates can help patients get appointments^21,24^ (R).
	• Speaking with asthma nurses is an effective way for patients to gain access to medical knowledge and ask questions that they might feel uncomfortable asking a GP^52^ (Ca). Patient advocates can help patients when they experience difficulties making appointments^21^ (P, R).
	• Some South Asian patients report having more difficulties accessing primary care during an attack than white patients^44^ (P).
	• Lack of access to specialist care is a potentially preventable factor that may lead to ED re-attendance^56^ (R).
Professional issues	• Issues within the healthcare system can affect provision of an action plan and guideline use. Issues include a lack of or limited health care resources such as, time restrictions during consultations, poor inter-professional communication between HCP and outside professionals, unclear roles, poor team work, and practical issues, such as access to lung function testing^39,41,43,46,54,69^ (HCP/P).
	• HCP’s and carers can be unaware of the limited availability of school nurses. School policies can be unclear as to how asthma is managed in a nurses’ absence^31^ (HCP/S/Ca). Communication is considered to be poor between HCPs, school nurses, parents and teachers^39^ (P/Ca/HCP).

^**a**^ Type of person who expressed their viewpoint (*P* patient viewpoint, *HCP* health care professional viewpoint, *Ca* carer viewpoint, *Ch*—child/adolescent’s viewpoint, *R* researcher’s viewpoint, *S* school personnel’s viewpoint)

**Table 7:** Barriers and facilitators to asthma self-management

Themes identified	Barriers	Facilitators
Partnership between patient and/or carer and HCP	• Patients feel there is poor communication between the patient and/or carer and HCP(P)	• Having patient advocates facilitates communication between the patient and HCP(P)
	• Patients perceive a lack of consistency in advice given to them by GPs, leading to confusion. (P)	• Communication between patients and HCPs where the patients feel they are being listened to (P)
	• Patients experience a lack of continuity of care. (P)	• Patients who have a mutually trusting relationship with their GP have confidence in their own understanding of asthma and are more likely to adhere to SM advice (P)
	• Patients (including ethnic minorities and low health literacy) sometimes do not perceive their treatment as effective, and therefore choose not to comply with treatment advice (P)	• Patients want GPs to take an interest in them and understand their experiences. (P)
	• Patients with intellectual disabilities are frustrated when HCPs talk to carers rather than them directly. (P)	• HCPs who are empathic, non-judgemental, and open. (HCP)
	• Young people and adolescents do not perceive the need for regular reviews. (P)	
Issues around medication	• Patient beliefs that asthma medications are unsafe, have side effects, and lead to long-term dependence and addiction. (P)	• Some patients use trial-and-error approaches in conjunction with their GP, which increases their confidence in managing their asthma (P)
	• Patients’ (including adolescents, ethnic minorities, and intellectual disabilities) reluctance to use medicines regularly, leading to them using preventative medicines inconsistently (P, C)	• Many patients use CAM to be in control of medication rather than feeling dependent on it. (P)
	• Patient reluctance to use reliever medication due to side effects or beliefs that their symptoms are not bad enough to warrant use (older patients) (P)	• Carers believe medicines are necessary (C)
	• Patients reluctance to follow GP treatment plans that include high doses of preventer medications, due to beliefs high doses are not useful (P)	• Strategies to remember when to take medications (for patients including African American adolescents and intellectual disabilities). Includes visual cues, reminders, setting phone alarms and matching to routine daily events. (P,C)
	• Patients who see the GPs plan as too drastic, adapt their own plan, based on their experience of dealing with symptoms. (P)	• A proactive attitude. (P)
	• Perceptions of PEF and inhaler use as too time-consuming. (P)	• Adolescents are more likely to use inhalers if it is nearby and can be used without disrupting activities, with sufficient privacy, and with support from friends, teachers and coaches. (P)
	• Problems accessing medications (P)	
	• Problems understanding medication instructions (including adolescents and older adults) (P)	
	• Not taking medicine as prescribed and not attending medicine reviews (P)	
	• Using a trial and error approach to taking medications, based on symptoms. This can cause symptoms to worsen, leading to the belief that preventative medications are not necessary (typically used by patients, including young adults and carers, who don’t believe they have asthma) (P)	
	• Preference for children to take a particular type of medication, based on habit, method of administration, confusion and fear of taking too many medications. (C)	
	• Carers sometimes skip doses to avoid upsetting their child (i.e., when trying to get to school on time). (C)	
	• Difficulties administering medication during school hours (C)	
	• Adolescents are reluctant to use inhalers if they have to leave a class or lack privacy. (P)	
Education regarding asthma and its management	• Lack of understanding of medications, low asthma knowledge, and lack of knowledge about measuring PEF (P, C)	• Asthma education can enhance adherence to treatment plans and help prevent ED re-admittance (P)
	• Low asthma knowledge, and lack of understanding of medications or use of an asthma action plan within schools. (C,S HCP)	• Patient advocates, pharmacist educators and nurses can help with SM education (P)
	• Teachers not believing adolescents when symptoms are reported. (P)	• Asthma camps can help those with severe asthma in learning about asthma management (P)
	• Education that is not adapted to individual needs. (P)	• Asthma education leads to good recognition of symptoms (P)
	• Information booklets seen as inadequate for some patients with severe asthma (P)	• Most patients actively avoid triggers to manage their asthma (P)
	• If patients are unable to recognise their symptoms, they do not use their asthma action plans (P)	• South Asians who were educated in using action plans became confident in and receptive to using them to manage their asthma (P)
	• Inability to identify triggers (P)	• Brief information and oral information from doctors tailored to the needs of the individual, and peer group support are the preferred method of education. (P)
	• Patients would like to be shown how to use their inhalers effectively (including learning disabilities). (P)	
	• Some patients find it difficult to distinguish their symptoms from a common cold (P)	
	• Many South Asians report poor understanding of their medications, and use fewer preventative medications and less corticosteroids during an attack than white patients (P)	
	• It is carers’ responsibility to educate teachers in asthma SM (C)	
	• Asthma nurses and GPs feel they have insufficient training in action plan use (HCP)	
	• Information obtained from potentially unreliable lay sources (including low health literacy, ethnic minorities, adolescents and carers)	
	• Those with a highly personalised sense of responsibility do not accept generalised advice and do not want to work in partnership with their GP (including African American adolescents). (P)	
Health beliefs about asthma and self-management	• Patients omit health beliefs that are not consistent with Western medication from discussions with their GP (P)	• Patients who have confidence in taking medicines avoid ED re-attendance. (R)
	• Not accepting the diagnosis (denying asthma or minimising its severity). This can mean patients forget medications and do not follow action plans. (P)	• Text message mobile technology can help some patients come to terms with their diagnosis, and can help monitor symptoms to aid transfer of responsibility (P)
	• Belief that the disease in only present when symptomatic (including ethnic minority carers). (C)	• Patients dislike the negative self-image associated with asthma and feeling out of control with their symptoms, which can motivate them to fight back (P)
	• Despondency following asthma attacks despite positive personal action. (P)	• Patients are motivated to manage their asthma when symptoms cause discomfort, if they believe it may have serious or life-threatening consequences, and when it affects a valued activity (P)
	• Being embarrassed to use inhalers in public due to concerns about what others will think (P)	• Those with high self-efficacy for asthma management believe that exercise, trigger avoidance, using an inhaler and preventer medication make a difference (P)
	• Adolescents do not always see the importance of seeing the doctor for review when feeling well, and are sometimes unwilling to take their medications (including African Americans) (P)	• Some children take responsibility for their asthma and are aware of their triggers (P)
	• Adolescents sometimes have worse morbidity due to less parental supervision (P)	• Carers want their children to be treated normally, and the asthma not limit their lives (C)
	• Patients who normalise their symptoms have no motivation to self-manage (P)	• Patients aim to live symptom-free or to have control over their asthma (P)
	• Those with limited perceived ability to control asthma have poor asthma control (P)	• Involvement of children in consultations so they can show their parents they are becoming independent (P/C/HCP)
	• Those with limited perceived ability to control external or environmental factors have poor self-efficacy for asthma management. (P)	• Some patients set goals to learn to live with asthma. (P)
	• Patients who believe self-management is their responsibility based on their own judgement and awareness do not perceive the need for an alliance with their GP. (P/C)	
	• Few patients have a goal, have worked with a HCP to set a goal, or have planned ways to achieve a goal. This is perceived to be due to a lack of time in consultations. (P/HCP)	
	• Patients and carers aim to treat symptoms (rather than prevent symptoms or attacks; includes African American adolescents and young adults) (P/C)	
	• Carers and children hold differing views on how to be responsible for asthma (P/C)	
	• Carers not accepting the diagnosis due to stigma and no diagnostic test (C)	
	• Carers adapt their written asthma action plan to focus on the most bothersome symptoms (C)	
	• Parents have concerns over balancing monitoring medication use and encourage medications (C)	
	• School staff are unclear how to manage asthma, and sometimes do not believe adolescents when they report symptoms (P/C/S)	
Self-management interventions	• Some patients do not have or have ever seen an action plan (P)	• Patients feel that regular reviews could facilitate their control and empower them to self-manage their condition. (P)
	• Adolescent patients not being willing to use electronic action plans (P)	• Cregivers instigate the development of an asthma action plan with school nurses (carers of African American adolescents) (C)
	• Patients see action plans as not relevant to them personally. (P)	• School nurses and staff want asthma action plans for all children (S).
	• GPs see action plans as not relevant for certain categories of patients (HCP)	• HCPs plan ahead if they are expecting a child who will need school documentation including an asthma action plan. (HCP)
	• GPs seeing action plans as irrelevant, impractical and time-consuming (HCP)	• Patients seeing action plans and guidelines as useful (P)
	• GPs not seeing guidelines as useful for medication adjustment (HCP)	• Patients seeing internet interventions as useful for identifying triggers, monitoring symptoms, feedback regarding lung function, and reaction to changes that occur in asthma status (including young African American patients) (P)
	• Nurses believing action plans are dangerous for some people (HCP)	• Patients feeling supported and in control being monitored by a text message diary or mobile phone alarm (including intellectual disabilities) (P)
	• Lack of confidence with computers can hinder use of internet interventions (P/HCP)	• Older Adults find it useful to create reminder systems and routines to facilitate medication use. (P)
	• Patients and GPs having low self-efficacy for using action plans (P/HCP)	• Written information booklets and videos in patients’ native languages would be useful (P)
	• Patients believe peak flow monitoring is nonsense or frightening. (P)	• A pharmacist telephone intervention was helpful for receiving SM education, asking questions and receiving feedback (P)
	• Mood and memory problems prevent the use of asthma action plans (African American Women). (P)	• Internet interventions were accepted better by those with poorly controlled asthma (P)
	• During regular asthma reviews, patients are sceptical about the interest, knowledge, and understanding demonstrated by GPs. (P)	• GPs seeing guidelines as useful (HCP)
	• Patients perceive generic action plans as patronising or condescending. (P)	• An Internet intervention led to improved understanding of asthma, reduced symptoms and improved compliance, and improved record keeping and performing calculations (HCP).
	• Patients deciding to follow their own plan of action without consulting their GP until they feel the need to. (P)	
Co-morbid conditions	• For patients with a co-morbidity, managing asthma may not be their top priority (P)	• Patients can engage in health lifestyles (e.g., weight loss) in order to benefit asthma and other conditions at the same time. (HCP)
	• Asthma medications can have an undesirable effect on other health conditions. (P)	
	• Patients may not adhere to asthma medication if they have too many other medications as well. (P)	
	• Comorbid conditions (e.g., mood/memory/pain) can constrain asthma management (e.g., by forgetting to take medications, or reducing physical activity). (P)	
	• Asthma symptoms and management can constrain the management of comorbid conditions (e.g., corticosteroids slowing weight loss). (P)	
Mood disorders and anxiety	• If patients are depressed, they may neglect SM (P)	
	• Anxiety about asthma can reduce the emotional wellbeing of older adults. (P)	
	• Other stressors (such as employment, housing issues, difficulties with personal relationships and fear of deportation) can lead to stress causing patients to neglect SM or contribute to exacerbations (P)	
	• Carers find it stressful managing their children’s asthma, which exacerbates their symptoms (C)	
	• Many families experience difficulty with the transition of responsibility from carer to patients (C/P)	
	• Patients are anxious about the possibility of having a severe asthma attack in public, with bystanders not knowing what to do. (P)	
Social support	• Family members can unhelpfully over or under react to asthma symptoms. (P)	• Patient advocates can provide social support to the patient (P)
	• Family members sometimes nag about medication taking and disregard severe symptoms or are unwilling to talk about the illness after an attack. Family members sometimes also warn patients of side effects of steroids, such as weight gain. (P)	• Family, friends and co-workers can remind patients to take medication, teach them about asthma, keep them calm during an attack, provide transport to appointments, not smoke inside the house, help with domestic duties when symptoms are increasing and provide emotional support (P)
	• Some patients do not comply with treatment if GP recommendations conflict with opinions of family and traditional healers (P)	• Patients would like local support groups. (P)
	• Employers may decline to employ those with asthma if there is a history of absenteeism due to their asthma. (P)	
	• Patients with asthma do not like to impinge on others’ lives. (P)	
	• Adolescents can be reluctant to tell their friends about their asthma and can experience social rejection by teachers and peers if perceived to disregard their asthma symptoms. (P)	
Non-pharmacological methods	• Patients and carers delay reliever medication use by attempting to manage asthma symptoms using non-pharmacological methods (e.g., drinking water, inhaling steam, resting, using a fan). (P/C)	• Preventative methods are used to attempt to avoid onset of asthma symptoms (e.g., acupuncture, use of a dehumidifier). (P/C)
	• Patients (adolescents) with uncontrolled asthma had greater use of non-pharmacological methods resulting in delays to medication use. (P)	• Lifestyle changes (e.g., diet weight loss, exercise) are used to attempt to improve asthma control (P)
Access to healthcare	• Patients are sometimes unable to access treatment due to costs of healthcare, insurance and problems accessing medications (P)	• Patient advocates can help patients get appointments (P)
	• Patients experience difficulties in getting appointments in primary care due to long waiting times (P)	• Patients find speaking with asthma nurses an effective way to gain access to medical knowledge and ask questions. (P)
Professional factors	• Healthcare professionals experience barriers to implementing guidelines and action plans. These include time restrictions during consultations, lack of support from outside professionals, unclear roles, poor teamwork, and lack of access to lung function testing (HCP)	
	• HCP’s and carers unaware of the limited availability of school nurses. (HCP/C)	
	• School policies can be unclear as to how asthma is managed in a nurses’ absence (HCP/S/Ca).	
	• Communication is considered to be poor between HCPs, school nurses, parents and teachers (P/Ca/HCP).	

The first theme, presented in Table 1, identified the need for a sense of partnership between the patient/carer and their healthcare professional. This theme was identified within only 26 (46%) of the included papers, but was expressed strongly in those papers. Facilitators identified by both patients and HCPs include the view that good communication based on mutual trust and respect gives patients and carers confidence in their understanding of asthma, and increases the likelihood of them adhering to self-management advice. Unfortunately, this was an area in which frustration was often expressed, and an absence of this partnership commonly reported, particularly by adolescents and young people, those with low health literacy or intellectual disabilities, and those from ethnic minorities. Patients and carers had specific expectations of their HCP, in relation to feeling listened to, being in partnership, and the need for consistent personalised advice and information. Indeed, a perceived lack of continuity in advice could lead to the belief that care and treatment is ineffective, and the decision not to comply with advice.

The next theme focused on patient and carer *issues around medications,* (Table 2) and was a dominant theme, reported within 39 (70%) of the included papers. Barriers, rather than facilitators, tended to be discussed within this theme, with 21 papers raising patient, child and carer concerns over the safety and side effects of asthma medicines. However, some studies did report facilitators in the form of strategies, particularly for teenagers and those with intellectual disabilities, who along with those from ethnic minorities, older adults, and other patients, tends to avoid ‘too much’ ‘toxic’ medication use due to fear of side effects, tolerance and addiction.

Other medication barriers included practical barriers, such as costs of medications, misunderstanding medication instructions and the inconvenience of remembering and administering medication, particularly for children and school staff during school hours. Some patients and carers experiment with action plans and timing and dosages of medication, which can cause symptoms to worsen. However, when done in collaboration with a HCP it can facilitate asthma self-management by increasing confidence. Some patients and carers had preferences for particular types of medication, including CAM use, which was considered mainly by women, and in combination with conventional medicines.

A need for more *education regarding asthma and its management* was also a dominant theme that was identified, being discussed in 40 (71%) of the papers (Table 3). With regards to barriers, many healthcare professionals feel they have insufficient training in action plan use. From the patient perspective, the understanding and awareness of asthma, asthma control and triggers, as well as an understanding of medication and appropriate use of medication appears to be a concern for most patients. This seemed to be relatively universal including among those with low health literacy or intellectual disabilities and those from ethnic minorities, who all tended to seek out information from lay sources. Some of the papers more closely explored how children and adolescents’ asthma is managed at school, with adolescents, carers and school staff all expressing a greater need for education, communication, and clearer processes. More concerningly, adolescents and their carers (particularly African Americans) reported that teachers sometimes did not believe the adolescents when they reported having asthma symptoms.

In relation to facilitators, education focusing on asthma self-management can improve asthma management and enhance recognition of symptoms, leading to reduced emergency department re-attendance. Interventions to improve education by using patient advocates, nurses and pharmacist educators have shown preliminary positive results in facilitating communication between healthcare professionals and patients, helping to obtain appointments for patients, providing social support to patients, and reinforcing self-management education. However, for acceptability it was important that the recipients felt that the education being offered was tailored to their needs. Needs were perceived by those with asthma to vary by age group, culture, language and ethnicity. Such education was reported to improve the use of action plans.

Information around how *health beliefs* influence self-management in patients with asthma are reported in Table 4. This theme was identified in 43 (77%) of the included papers. Beliefs about asthma can motivate very different behaviours. For example, some find poorly controlled asthma to be embarrassing, stigmatising and burdensome, so they try to conceal or normalise their symptoms or they may not take their medications or follow action plans. By contrast, others respond in a way that facilitates motivation to learn to live with their asthma, and fight back and gain control by taking their medications so they can engage with their everyday activities and prevent further attacks.

Barriers surrounding the sharing and transfer of responsibility between adults/carers and HCPs, as well as between children, their carers, and school staff with regards to asthma management also raise a range of different issues which, if not carefully addressed, can commonly result in confusion, disagreement and mismanagement. With regards to facilitators, nurses believe that involvement of children in consultations can facilitate self-management, as it provides an opportunity for children to show their parents they are becoming independent.

Feedback from healthcare professionals and patients regarding *self-management interventions* were reported in only 27 (48%) of the papers (see Table 5), but like the first theme, views in this theme were strongly expressed. Interventions included use of action plans, guidelines, internet and text message interventions to improve aspects of self-management; educational interventions in the form of a booklet or DVD; and medication reviews. Within this theme a greater balance of barriers and facilitators were expressed than for other themes. The main facilitator to asthma self-management was if healthcare professionals and patients regarded action plans and guidelines as useful. However, among those who seemed only marginally positive about action plan use, there seems to be an ‘ideal’ person for whom action plans were suitable, which often did not include themselves or their patients. Conversely, if generic action plans were used, or if healthcare professionals had negative views about action plans, the quality of their relationships with patients was reduced. Just as some healthcare professionals have reservations about guidelines and action plan use, preferring to rely on their own judgement about how to treat patients, some patients also felt this way about managing and modifying their own asthma care without consulting their healthcare professional.

Both healthcare professionals and patients/carers responded positively to the use of technology (mobile phone alarms, text messaging, emails, internet) to monitor and encourage self-management, provided they were familiar with using e.g., computers, mobile phones and systems did not take too long to access. These technological interventions were particularly valued by those with intellectual disabilities, adults and older adults, however, a lack of confidence with computers was one of the main barriers to using online self-management interventions for both patients and HCPs alike. Technology was also valued by patients, particularly young patients and those with poorly controlled asthma, to monitor their symptoms as part of an internet-based electronic action plan. Patients valued self-management education from a range of sources provided the style of writing was appropriate and comprehensible to lay people, and in their own language.

The last six themes are presented in Table 6. These themes occurred much less frequently than the first five themes. The presence of *co-morbid physical conditions* (discussed in 5 (9%) of included papers), can be a barrier to asthma self-management if the management of the different conditions conflict, and if asthma is not the patient’s top priority. However, healthy lifestyle behaviours (e.g., weight loss) were seen to facilitate benefit to multiple conditions at the same time.

Having a *mood disorder or anxiety* was reported in 10 (18%) papers. Carers and families often find managing the child’s asthma stressful, and may pass their worries on to the child. Many families experience stress around the transfer of responsibility from carer to child. Stressors or depression may also contribute to exacerbations or cause patients to neglect self-management.

The amount and type of *social support* patients have access to can act as facilitators or barriers to asthma management. Social support can have both *positive influences* (reported in 16 (29%) of papers), including friends and family members reminding those with asthma to take their medication, and by providing practical and emotional support. However, *negative influences* and barriers were also reported, within 13 (23%) of included papers, where friends or family members upset those with asthma by perceived over- and under-reactions to the condition such as disregarding severe symptoms, or giving opinions that conflict with GPs advice. This was particularly the case among ethnic minorities, and can lead to patients not complying with recommended treatment.

Patients with asthma use a variety of *non-pharmacological methods* in primarily three different ways to facilitate self-management. This theme was discussed within 12 (21%) of papers. In many cases methods such as drinking water, resting, or inhaling steam were used to try to relieve early asthma symptoms before taking reliever medication. Methods such as acupuncture or regularly opening windows were used in order to avoid the onset of asthma symptoms. Lifestyle changes (such as weight loss and exercise) were used with the aim of improving asthma control.

Issues involving *access to healthcare* (reported in 12 (21%) of the papers), can impact on the patient’s perceived ability to self-manage their asthma. Some patients have reported difficulties in accessing healthcare, including problems getting appointments in primary care, costs of healthcare, insurance and problems accessing medications. To facilitate access to healthcare and therefore self-management, patient advocates can help patients overcome access issues and asthma nurses can provide information about asthma management, particularly when GP appointments are not possible.

Finally, *professional issues* were reported in 7 (13%) of the papers. Only barriers were raised in this theme. Professional and organisational factors such as time restrictions during consultations, poor role definition and levels of teamwork and inter-professional communication, as well as practical issues such as access to testing, can act as barriers to implementing action plans and guidelines. Within schools, a lack of clarity regarding policies relating to asthma management, and poor communication between HCPs, school nurses, teachers and parents are also barriers to effective self-management.

## Discussion

### Main findings

This review aimed to identify individual patient, professional and organisational barriers and facilitators to asthma self-management, by examining qualitative evidence from the perspectives of patients, carers and healthcare professionals. Eleven themes were identified: partnership between the patient and their healthcare professional; issues around medications; education regarding asthma and its management; health beliefs; self-management interventions; co-morbid conditions; mood disorders and anxiety; social support; non-pharmacological methods; access to healthcare; and professional factors. Within the themes, barriers and facilitators at the level of patient, carer and healthcare professional were identified, which are summarised in Table 7.

### Interpretation of findings in relation to previous research

Previous reviews have highlighted the importance of good communication between the healthcare professional and patient.^19,20^ The findings within our first theme, on partnership between patients and HCPs, were consistent with the findings of the previous reviews. Being able to listen to and respect patients was conducive to effective action plan use, and patients valued a healthcare professional who was sensitive and empathic. Patients and healthcare professionals also described aspects of asthma in different ways, and patients (particularly those with severe asthma) reported that information given by their healthcare professional should be easily understood. When patients view their treatment as effective, the patient/healthcare professional partnership is strengthened. One previous review^19^ suggested that the extent to which the patient was agreeable to advice and management strategies was determined by the ‘likeability’ of the provider. Our review adds that seeing the same GP across visits and receiving consistent advice also contributes to adherence.

Patient concerns regarding the safety of inhaled steroids and fears of dependence upon asthma medications have also been raised in previous reviews.^19,20,76^ Patients have reported that they experimentally adjust their medications and adapt the advice given by their healthcare professional, and reduce their dosage particularly when symptoms have reduced. Practical barriers to medication adherence, such as costs and the inconvenience of having to remember to take medications, have also been identified previously. In addition, our findings report the patient experience regarding dangers of such trial and error approaches to medication taking, and that patients use CAM in response to fears about more traditional asthma medicines. Our findings also suggest that the use of cues, technology based reminders, and routines may be facilitators, particularly for ethnic minorities and those with intellectual disabilities.

Patients have previously reported difficulty in recognising symptoms, not understanding what an asthma attack actually is, and having inadequate knowledge about asthma and its treatment.^19,20^ We concur with these findings and conclude that education is needed to help patients identify triggers. We also found that healthcare professionals perceive other barriers to asthma education, such as language barriers and poor understanding of medications, which may be more pronounced with ethnic minority patients. These concerns indicate changes are required to improve their engagement with the healthcare system.^77^

We found that when patients believe their asthma is serious, they are more likely to adhere to self-management strategies. However, perceived stigma regarding asthma leads to reduced adherence to medications, particularly in public. Similar findings have been reported regarding medication adherence and negative views of asthma.^19,78^ Our review also revealed that perceived negative views of asthma have led older people to conceal symptoms. These findings emphasise the importance of patient acceptance of asthma diagnosis and how this influences asthma management. This has been reported in relation to successful management in other conditions.^79–81^

Text message, Internet, booklet, DVD, and pharmacist interventions were perceived as acceptable self-management programmes. When consulted, patients report ways to improve interventions, which in turn might lead to improved self-management adherence. However, findings regarding action plan and guideline use are consistent with evidence that both healthcare professionals and patients hold negative views regarding the usefulness and practicality of action plans and guidelines, believing they are useful only for some people.^20^ Incompatibilities between reviews were identified regarding action plans, because the action plans were carried out on different patient populations. An Asthma UK report on barriers to effective emergency asthma care also recommended promoting awareness of guidelines by “signposting” patients to charities and discusses internal (negative beliefs regarding the suitability and evidence base of guidelines) and external (financial incentives or penalties) motivators of guideline use.^82^ The latter refers to the commissioning for quality and innovation framework (CQUIN) in the UK, where a proportion of healthcare providers’ income was linked to achieving good, quality practice.

Issues not raised in previous reviews were patient reported mood and anxiety problems that can impair self-management, and having a comorbidity perceived by patients as more important to treat.^19,20,82^ One previous review highlighted positive influences of social support,^19^ but we found it can also impact negatively on self-management outcomes, including over-and under-reactions to the condition by family members, and employer concerns regarding absenteeism due to asthma. Research in our review identified that the use of non-pharmacological methods to delay medication use appears to be contributing to poor control. Perceived access to healthcare is also raised in this review and highlights the use of patient advocates to assist with issues such as difficulty getting appointments.

### Strengths and limitations of this review

This review includes mainly Caucasian patients with asthma, although some studies have explored the views of minority ethnic and other at risk groups. The majority of the issues uncovered still need to be explored further in these subgroups. Also, although we used a wide range of search terms, we may not have identified all published qualitative studies. Overall, the quality of the reviewed studies was high. However, future studies should provide sufficient information to enable assessment of whether the researchers have adequately considered the relationship between researcher and participants, and whether ethical issues have been considered.

### Implications for future research, policy and practice

There are several areas that could impact on future research, policy and practice. With regard to practice, better adherence to asthma self-management may be achieved in a number of ways. First, evidence from our review suggested that educational interventions (including mobile phone and internet interventions) facilitated asthma self-management, and were perceived as acceptable and useful by HCPs and patients. Therefore, some patients, carers, teachers and healthcare professionals may benefit from further education. However, as some patients lack confidence with computers, their skills should be assessed prior to referral to ensure they receive appropriate interventions. With regard to research, future self-management intervention trials aiming to increase adherence to medications and action plans should include tailored education on recognising asthma symptoms, triggers, how to recognise an attack, patient concerns and beliefs regarding medications and non-pharmacological methods; and the importance and necessity of preventative medicine. Patients should also be advised to adjust medication only in collaboration with their GP, as a trial and error approach can cause symptoms to worsen.

The second area relates to the partnership that is built up between the healthcare professional and the patient/carer. A good relationship between the healthcare professional and the patient or carer facilitates asthma self-management. In practice, healthcare professionals should aim for continuity of care, so they are able to give consistent advice, be aware of the history, background (mental health and co-morbidities), and personal circumstances of the patient (such as social support networks). They should also try to understand the beliefs the patient and carer hold about asthma and their medications, as negative beliefs about medication may act as a barrier to effective self-management, and can be addressed if brought up in consultation.

Third, some GPs had negative views regarding the usefulness of guidelines and action plans, so did not always use them to conduct evidence-based practice. This is a modifiable barrier to asthma self-management. The use of proformas to ensure patients undergo care that follows current guidelines has been suggested,^1^ and we concur with Asthma UK’s recommendation of providing training to healthcare professionals to enhance feelings of competence in implementing guidelines.^82^

The fourth area is concerned with the professional and demographic features of the deliverer of the intervention. Evidence from this review suggests that nurses and AHPs are considered an effective source of information, so facilitate self-management. They could potentially be used instead of or in addition to doctors to deliver self-management interventions. Our review also provides evidence that pharmacist and patient advocate interventions facilitated asthma self-management.^21,25^ Future research could examine whether these or other healthcare professionals (such as physiotherapists) can deliver interventions to achieve outcomes as efficiently as nurses and doctors, and clarify the most effective team-based approach.

Fifth, cultural factors should be researched. Our findings indicated that some Latino, African American, and South Asian patients perceive access to healthcare as an issue, and have poor understanding of their medications. However, education in using action plans increased confidence in some of these populations. These findings suggest that ethnic minority patients may need more tailored education to facilitate understanding of their medications. Future qualitative research is required to explore how barriers to effective self-management might differ according to ethnic background, and whether separate interventions presented in the patients’ own language, and involving the family would benefit patients.

The sixth area for future investigation is the age of the person with asthma, as different factors might influence intervention success in older and younger populations. Only one study specifically explored older patients’ (≥50 years) beliefs regarding asthma. Wider research suggests older patients are at risk of being non-compliant in taking medications.^83^ Our findings suggest that this is the case for older patients with a longer-term diagnosis, who tend to have poorer asthma control. They may benefit from interventions focusing on education regarding acceptance of their condition and issues around medications. With regard to younger patients, this review suggests that in practice, involving children and adolescents in consultations, to show their carers how independent they are, could help facilitate the transfer of responsibility from the carer. As adolescents do not always take their asthma medications or attend asthma reviews, research could develop educational interventions (possibly technological) about the importance of this. As carers often have to educate schools in asthma management, further research could address ways to educate teachers and peers in asthma management, to reduce the social rejection felt by some adolescents, due to their teachers and peers disregarding their asthma symptoms.

Finally, future research could examine whether the following factors impact on the outcome of self-management interventions: patient comorbidities (which are often neglected in research on chronic disease), mood disturbances or anxiety, and the impact of the patient’s social support networks (which can act as a positive or negative influence on asthma self-management). The benefits and limitations of non-pharmacological methods to manage asthma could be further explored, and lastly, changes in practice could improve organisational barriers to asthma self-management, including time restrictions during consultations; support between healthcare professionals; unclear roles; poor teamwork; and practical issues such as lack of access to testing.

## Conclusions

This review identified barriers and facilitators to asthma self-management that might explain, in part, why existing self-management interventions are not always effective. The beliefs and motivations of each patient need to be explored to uncover any potential barriers that will prevent successful self-management. Improvements to self-management might be achieved by educating patients and healthcare professionals to alter current beliefs about asthma management that oppose effective self-management, and improving patient/healthcare professional relationships via training healthcare professionals in effective communication skills. By exploring perspectives from adults, children and carers, and healthcare professionals regarding factors that hinder their use and recommendation of self-management advice, organisational and structural issues have also been highlighted which interact to prevent the implementation of self-management. These, along with viewpoints from particular subgroups can help us to refine interventions, improve adherence and ultimately achieve better outcomes for people with asthma.

## Methods

### Search strategy

We aimed to conduct a systematic, inclusive, reproducible and extensive search, since qualitative synthesis benefits from wide sampling of the literature.^84^ Our search was carried out for the period between January 1996 and March 2017. Five electronic databases were searched (Medline, EMBASE, AMED, CINAHL, and PsycINFO), and we checked the British Thoracic Society Guidelines.^1^ Search strategies were developed based on MeSH terms. The thesaurus term ‘asthma’ was combined with either ‘self-care’ or ‘self-management’, depending on the database searched. Identified studies were then limited to those that included variations of qualitative research in the title or abstract (qualitative, ethnography, ethnographic, grounded theory, constant comparative/ comparison, content analysis, or thematic). One reviewer (CM or SK) screened the titles and abstracts against inclusion criteria, and the full texts of all potentially relevant articles were obtained.

### Inclusion and exclusion criteria

Included studies used qualitative data collection and analysis to identify perspectives of adults and children (and their carers) diagnosed with asthma, and the perspectives of healthcare professionals who were involved in providing interventions to improve self-management. Individuals with COPD were excluded. Non-English language studies, studies without any evaluative component, conference abstracts, PhD and Masters’ theses were excluded. Asthma self-management interventions were included if they used asthma education, self-monitoring, and/or the use of an asthma plan (digital or handwritten).

### Quality appraisal

The Critical Appraisal Skills Programme (CASP) appraisal tool for qualitative research was used to assess study quality.^85^ The tool asks 10 questions to assess the validity, relevance and results of findings. In line with best practice,^86^ 50% of papers were rated by two authors (CM or SK, and EAC). Minor discrepancies were discussed and resolved. Major discrepancies were resolved in discussion with one other author.

### Data extraction and synthesis

The results and discussion sections of the included studies were read through by one of the reviewers (CM or SK), to extract the findings. Data were analysed using thematic synthesis, following the process described by Thomas and Harden^87^ to organise and summarise the findings from the multiple qualitative studies identified.^84,88^ Text labelled as ‘results’ or ‘findings’ in papers were considered as review findings. In some places findings were reported in discussion sections and were therefore also included. Data were entered into NVivo software for qualitative data analysis. Thematic synthesis took place in three stages. First, initial codes were generated using line-by-line coding (using a word or phrase to describe what was happening in each line) and organised using NVivo software.^89^ New codes were developed throughout initial coding. Before completing this stage, all coded text were examined to check they had been interpreted consistently. In the second stage, codes were collated and organised into descriptive themes. Finally, themes were revised and re-grouped into analytical themes (initial themes were combined, separated and discarded) by group discussion among the researchers. Barriers and facilitators were inferred from the views expressed by patients, carers, and healthcare professionals, and the implications of these views for policy and practice were considered.
